# Supplementary material for: Neurospora sp. Mediated Synthesis of Naringenin for the Production of Bioactive Nanomaterials
Source: Bioengineering (Basel). 2024 May 18;11(5):510. doi: 10.3390/bioengineering11050510 (PMC11117806; doi:10.3390/bioengineering11050510)
Supplement: Supplementary file 1 [file bioengineering-11-00510-s001.zip › bioengineering-2906949-supplementary.pdf]

bioengineering

MDPI

Article

Neurospora sp. mediated Synthesis of Naringenin for the production of Bioactive Nanomaterials

Jitendra Dattatray Salunkhe [1], Indra Neel Pulidindi[2,*], Vikas Sambhaji Patil [3]and Satish Vitthal Patil [1,*]

[1] School of Life Sciences, KavayitriBahinabai Chaudhari North Maharashtra University, Jalgaon 425001, India; satish.patil7@gmail.com
[2] Jesus' Scientific Consultancy for Industrial and Academic Research (JSCIAR), Tharamani 600113, India
[3] University Institute of Chemical Technology (UICT), KavayitriBahinabaiChaudhari North Maharashtra University, Jalgaon 425001, India; vikasudct@gmail.com
* Correspondence: satish.patil7@gmail.com (S.V.P.); indraneelp@jesusconsultancy.com (I.N.P)

1. High-performance liquid chromatography (HPLC) analysis

Naringinase mediated naringin bio transforming product, i.e., naringenin was confirmed by using HPLC (Agilent Tech. Gradient System) with AGILENT- C18 column (4.6 x 250 mm, Particle size of packing 5 mm). The mobile phase comprised of methanol 60 % and 0.1 % formic acid 40 % at 26 °C temperature with a flow rate of 0.8 mL/min. A detection wavelength of 280 nm was used. The HPLC chromatograms showed the peak with the retention time of 3.4 min for the standard substrate (Naringin) (Figure S1.a); likewise for the biotransformed product (Naringenin) a peak with a retention time of 7.4 min. was observed. The peak at a lower retention time (Figure S1 b) indicated the unused substrate by the naringinase enzyme (Figure S1.b).

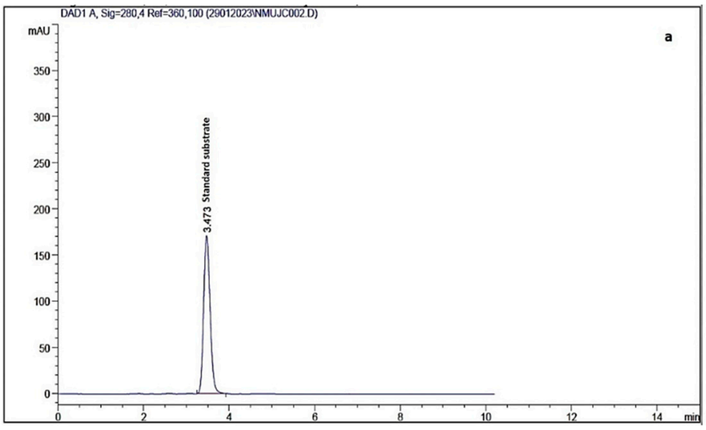

(a)

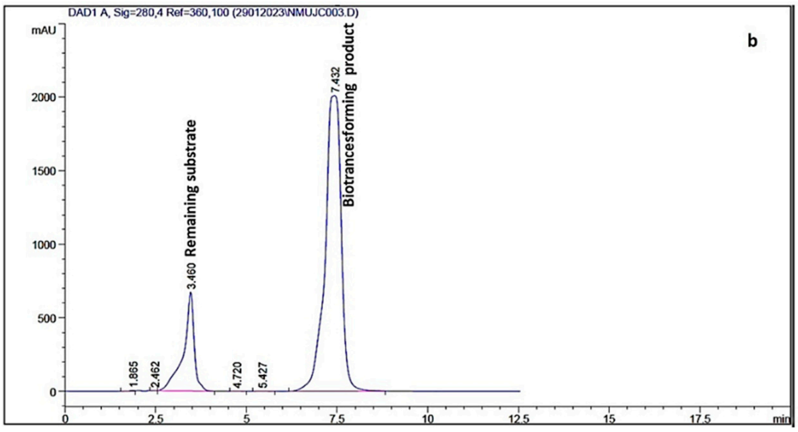

(b)

Figure S1. The HPLC chromatograms of the;**(a)** standard substrate (Naringin) and **(b)** naringinase biotransformed product (Naringenin).

2. Enhancement of naringinase activity with time

Table S1. Naringinase activity fold increases/decreases with incubation time.

Time (h)	Naringinase Activity (IU/mL)
24	30 ± 10.81
48	112 ± 3.60
72	285 ± 4.04
96	389 ± 4.04
120	386 ± 8.73
144	371 ± 7.54

Note*: Results are the mean of the triplicates; data represented here is in the form of mean ± SD.

3. Particle size distribution and zeta potential valules of Ag-naringenin nanoconjugates and Au-naringenin nanoconjugates

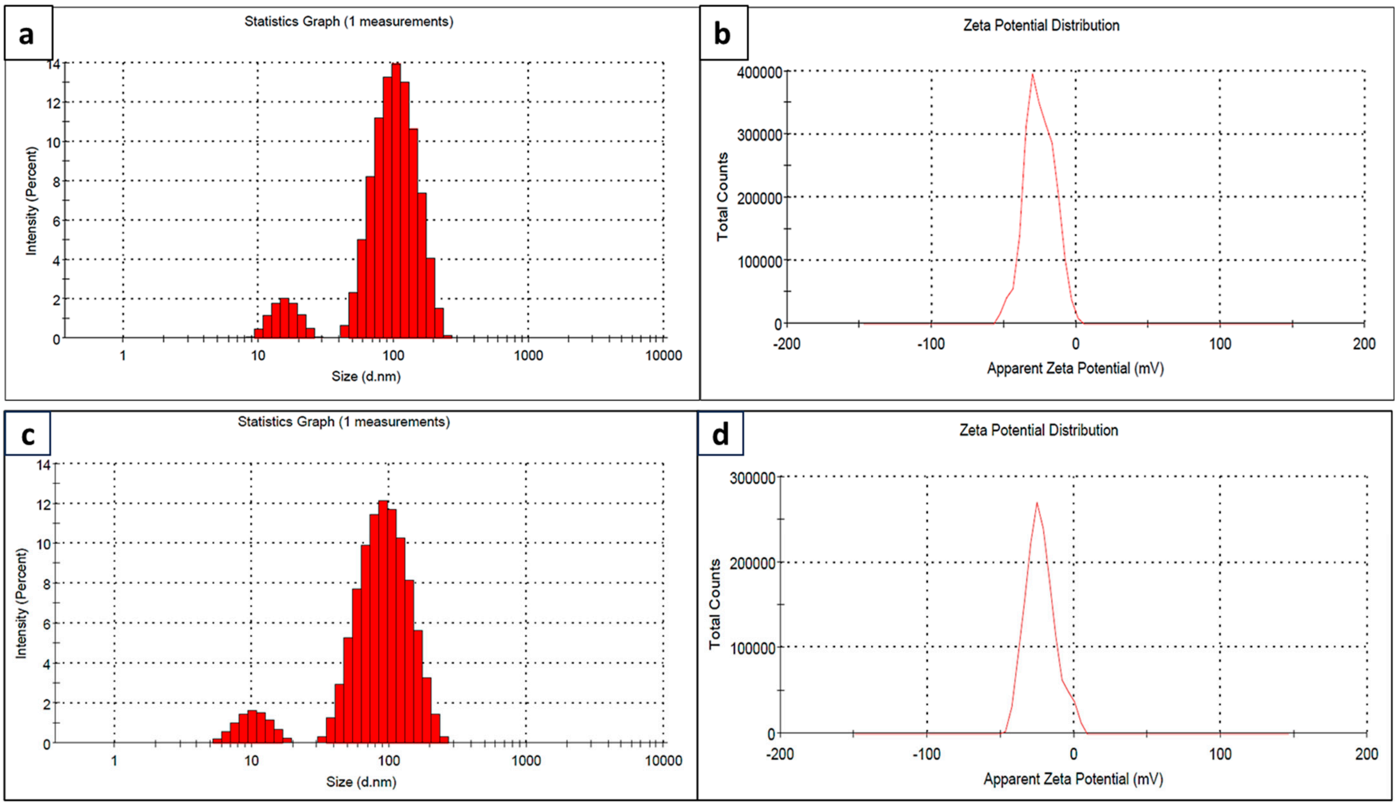

Figure S2. a) Particle size distribution plot of Ag-naringenin nanoconjugates (the average particle size of Ag-naringenin nanoconjugates is 80.82 nm); (b) Zeta potential of Ag naringenin nanoconjugates; (c) Particle size distribution plot of Au-naringenin nanoconjugates (the average particle size of Au-naringenin nanoconjugates is 65.25 nm) and (d) Zeta potential of Au naringenin nanoconjugates.

4. FT-IR analysis of naringenin, naringenin-Ag and naringenin - Au nanocconjugates

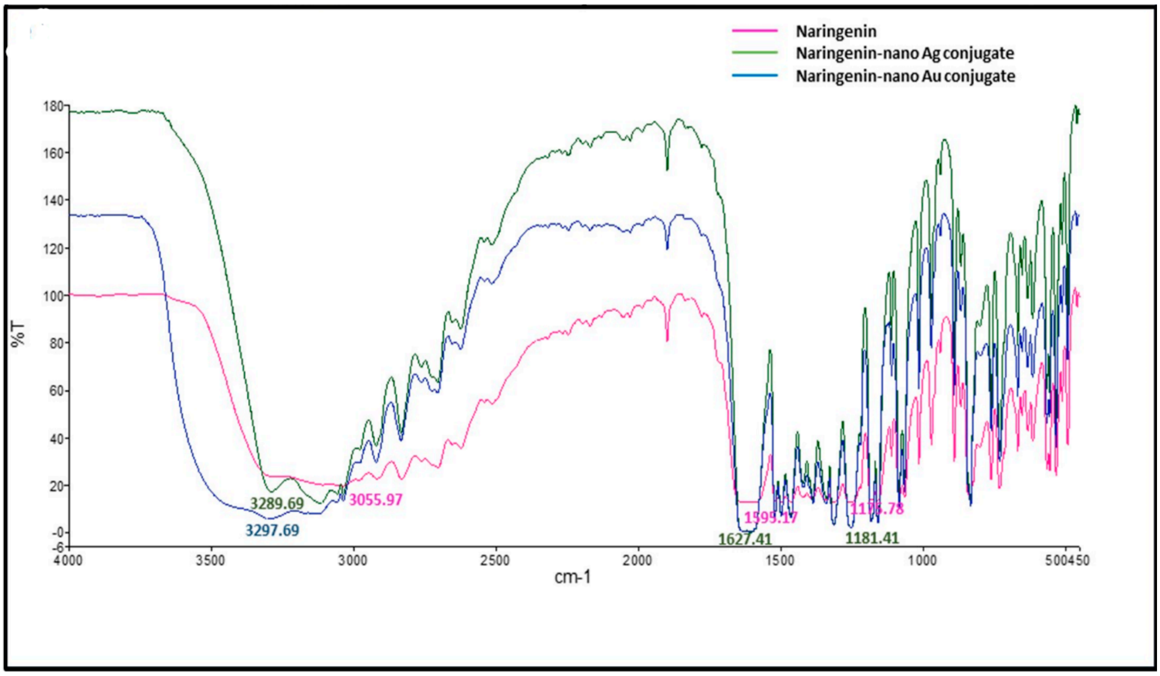

Figure S3. FT-IR spectra of naringenin, naringenin-Ag and naringenin - Au nanoconjugates.

5. Antibacterial activity – determination of the minimum inhibitory concentration (MIC) and minimum bactericidal concentration (MBC)

5.1. Materials and methods:

The lyophilized naringenin and the Ag-Nar conjugate's stock were prepared and used to prepare further dilutions i.e., seven different concentrations of 10, 5, 2.5, 1.25, 0.625, 0.312 and 0.156 mg/mL. The standard liquid medium dilution method as per CLSI M07-A8 was used for the study i.e., the antimicrobial activity was evaluated by visual observations of microorganism growth. The test compounds were serially diluted two-fold, with concentration ranging from 10 to 0.156 mL, and adjusted with test bacterial suspension i.e., *S. aureusATCC 6538* and *E. coliK12* (10^8 CFU/mL, 0.5 McFarland's standard) as representative examples and incubated for 24 h at 37 °C. The control consisted of only inoculated broth without the test compound. The minimum inhibitory concentration (MIC) determines the lowest concentration which does not show the turbidity visually. The MBC was determined by streaking a loopful of growth from all that tubes that don't show the visual turbidity on nutrient agar for 24 h at 37 °C. The lowest concentration showed no growth was considered as MBC [30,31].

Results:

S N	Organisms	MIC Naringenin mg/mL	MBC Naringenin mg/mL	MIC Nar-Ag mg/mL	MBC Nar-Ag conjugate mg/mL
1	*S. aureus ATCC 6538*	1.25	1.25	0.312	0.625
2	*E. coli K12*	1.25	1.25	0.312	0.625

Note*: MIC = Minimum inhibitory concentration; MBC = Minimum bacterial count; Nar-Ag = Naringenin-Silver nanoconjugate.

The Au-Nar conjugates does not show any significant antimicrobial potential. The biotransformed product naringenin has its minimum inhibitory concentration (MIC) and minimum bactericidal concentration (MBC) determined to be the same: 1.25 mg/mL for both gram-positive and gram-negative bacteria. The antimicrobial potential of the Ag-Nar

conjugates is also confirmed by determining their MIC and MBC in broth studies with gram-negative and gram-positive test organisms. It was revealed that Nar-Ag showed a MIC of 0.312 mg/mL and an MBC of 0.625 mg/mL, with similar values observed for both organisms, namely, *S. aureus ATCC 6538* and *E. coli K12*. These results agree well with the previous report on silver nanomaterial [30].
